# Sensitization of Antibiotic-Resistant Gram-Negative Bacteria to Photodynamic Therapy via Perfluorocarbon Nanoemulsion

**DOI:** 10.3390/ph15020156

**Published:** 2022-01-27

**Authors:** Peiyuan Niu, Jialing Dai, Zeyu Wang, Yueying Wang, Duxiang Feng, Yuanyuan Li, Wenjun Miao

**Affiliations:** School of Pharmaceutical Sciences, Nanjing Tech University, Nanjing 211816, China; niupy@njtech.edu.cn (P.N.); daijl@njtech.edu.cn (J.D.); wzyd@njtech.edu.cn (Z.W.); wangyueying@njtech.edu.cn (Y.W.); fengdx@njtech.edu.cn (D.F.)

**Keywords:** photodynamic therapy, antibiotic-resistant, oxygen-delivery, Gram-negative bacteria, sensitization

## Abstract

With the merits of excellent efficacy, safety, and facile implementation, antibacterial photodynamic therapy (APDT) represents a promising means for treating bacterial infections. However, APDT shows an unsatisfactory efficacy in combating antibiotic-resistant Gram-negative bacteria due to their specific cell wall structure. In this work, we report a perfluorocarbon nanoemulsion (Ce6@FDC) used as a multifunctional nanocargo of photosensitizer and oxygen for sensitizing antibiotic-resistant Gram-negative bacteria to APDT. Ce6@FDC was fabricated via ultrasonic emulsification with good colloidal stability, efficient Ce6 and oxygen delivery, and excellent photodynamic activity. Meanwhile, Ce6@FDC could strongly bind with Gram-negative *Acinetobacter baumannii* (*A. baumannii*) and *Escherichia coli* (*E. coli*) via electrostatic interaction, thus leading to notable photodynamic bactericidal potency upon irradiation. In addition, oxygenated Ce6@FDC also exhibited a remarkable efficacy in eradicating Gram-negative bacteria biofilm, averaging five log units lower than the Ce6 group under identical conditions. Taken together, we demonstrate that photodynamic perfluorocarbon nanoemulsion with oxygen-delivery ability could effectively kill planktonic bacteria and remove biofilm, representing a novel strategy in fighting against antibiotic-resistant Gram-negative bacteria.

## 1. Introduction

Antibiotics play an important role in the treatment of various pathogenic microbial infections. However, due to the abuse of antibiotics, the spread of antibiotic-resistant bacterial strains has arisen as one of the most worrying threats to public health in recent years, such as *E. coli*, *Staphylococcus aureus*, *Pseudomonas aeruginosa*, and *A. baumannii*, etc. [1,2,3]. These antibiotic-resistant bacteria could invalidate the primary treatment of traditional antibiotics through precise mutations, efflux pump expression, or up-regulation of defense-associated enzymes, and so on [4,5]. Although several dozen antibiotics are now in the R&D pipeline, most are modifications of existing antibiotic classes, for which progress is time-consuming and capital-demanding. Moreover, it is just a matter of time for microorganisms to develop resistance to these newly discovered antimicrobial agents, deriving from their single mode of action. Thus, the speed of new antibiotics development is far slower than that of multidrug-resistant bacterial evolution [6,7,8]. In addition, the formation of biofilm composed by Gram-negative bacteria and extracellular polymeric substances further results in high antibiotic resistance by restricting antibiotic penetration and inducing antibiotic inactivation [9,10]. Therefore, there is a pressing need to develop novel and effective therapies for combating bacterial infections and reducing the burden of antibacterial resistance via mechanisms different from those of traditional antibiotics in addition to the ongoing research to discover new antibiotics.

Antibacterial photodynamic therapy (APDT) is a promising alternative to antibiotic therapy because it induces few side effects, realizing precise treatment with outstanding biocompatibility [11,12]. APDT uses light at appropriate wavelengths to excite photosensitizers (PSs) to generate cytotoxic reactive oxygen species (ROS), predominantly singlet oxygen (^1^O_2_), to kill microbial pathogens. The generated ^1^O_2_ can damage membrane lipids, proteins, and cellular DNA, which involves a variety of cell death mechanisms. Furthermore, bacteria can hardly develop resistance to APDT due to it causing irreparable damage to multiple targets in accordance with the positioning of PSs in bacteria, which means it has a highly effective bactericidal effect and eliminates bacteria completely before they can gain anti-oxidative damage mechanisms [11]. However, it has been reported that there is a basic distinction in the susceptibility to PDT for Gram-positive and Gram-negative bacteria because of their inherent cell wall structural differences. PSs can relatively easily penetrate through the cytoplasmic membrane of a Gram-positive bacterial cell by a relatively porous layer of peptidoglycan and lipoteichoic acid and thus effectively kill the bacteria upon light irradiation, whereas the outer membrane of Gram-negative bacteria forms a physically and functionally defensive barrier to numerous drug molecules, which greatly hampers the efficiency of APDT [13,14,15]. Worse still is the anoxic environment resulting from the insufficient O_2_ supply, which is necessary in mediating the generation of cytotoxic ROS during APDT, further limiting the therapeutic effect of the oxygen-dependent PDT on Gram-negative bacteria.

Theoretically, the sensitivity of Gram-negative bacteria to PDT can be immensely improved via supplementing oxygen during the photodynamic process and relieving the anoxic or hypoxic environment. Recently, great efforts have been made to find a way out of this dilemma and to achieve a highly efficient APDT outcome for Gram-negative bacteria using organic or inorganic nanomaterials with oxygen- or ^1^O_2_-generation functionalities [16,17]. For instance, transition metal oxides, especially nanostructured MnO_2_, can react with endogenous hydrogen peroxide (H_2_O_2_) and H^+^ to generate oxygen, which could relieve hypoxia circumstances. Nevertheless, the use of metal ions might raise potential concerns over toxicity [18,19,20]. Perfluorochemicals (PFCs), a kind of inert organic compound, have been extensively explored as blood substitutes for tissue oxygenation, owing to their high affinity to oxygen and excellent biocompatibility [21,22,23,24]. Thus, it is rational to hypothesize that photodynamic nanomedicine with oxygenation ability using PFCs might be preferable for the sensitizing of antibiotic-resistant Gram-negative bacteria to conventional APDT.

**Figure 1:**
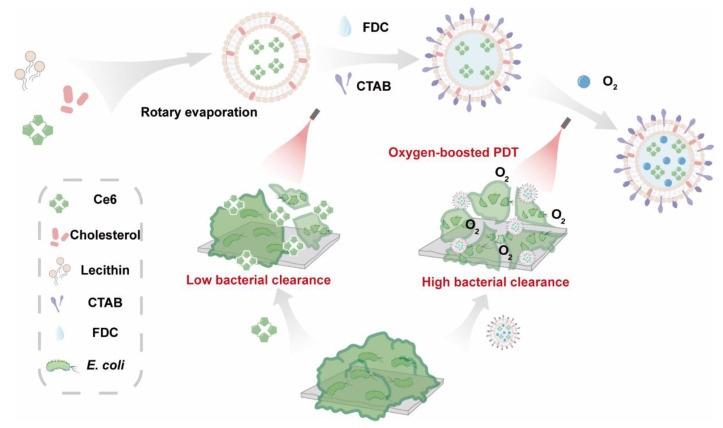
Schematic illustration for the fabrication process of Ce6@FDC nanoemulsion and its oxygen delivery for enhanced photodynamic antibacterial efficiency.

Inspired by the success of the oxygen-delivery strategy for the empowering of antibiotics and APDT [16,25], in this work, we propose an oxygen-affordable photodynamic nanomedicine (denoted Ce6@FDC) for the sensitizing of Gram-negative bacteria to APDT, especially clinically related drug-resistant strains (Figure 1). Among PFCs, perfluorodecalin (FDC), with its preeminent oxygen dissolving capacity and stability, was used here. Thus, Ce6@FDC, with the merits of low cost and ease of fabrication, is designed for oxygen delivery to increase the productivity of ^1^O_2_ for mitigating or overcoming the inertia of Gram-negative bacteria to APDT. With a suitably positively charged surface and high oxygen-carrying capacity, Ce6@FDC is able to effectively associate with bacteria and release oxygen, thereby empowering the efficacy of APDT. We systemically evaluated the oxygen-delivery behavior and photodynamic effect of Ce6@FDC. Following that, the antibacterial potency of Ce6@FDC + O_2_ on antibiotic-resistant *A. baumannii* and *E. coli* was investigated. By designing a late-model oxygen-delivery system, the sensitivity of Gram-negative bacteria to APDT could be enhanced comprehensively, which provides a strategic reference for the development of new antimicrobial agents.

## 2. Results and Discussion

### 2.1. Preparation of Ce6@FDC

**Figure 2:**
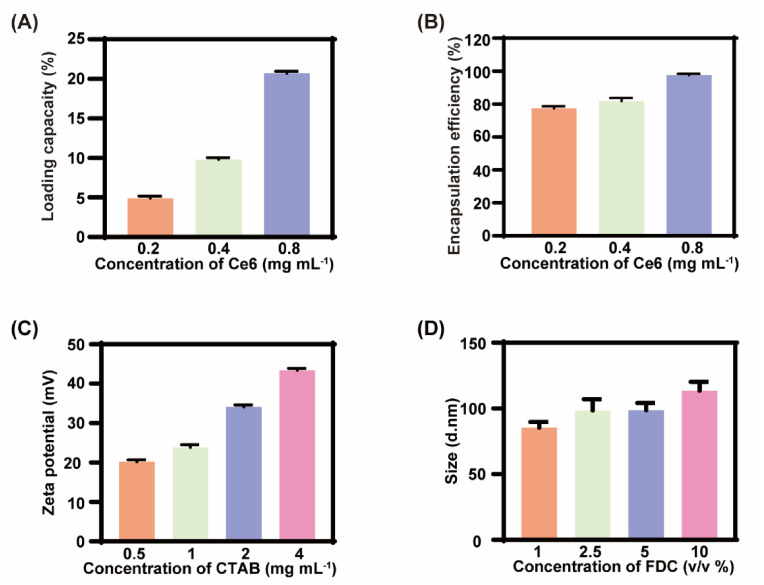
Formulation optimization of Ce6@FDC nanoemulsion. (**A**) Drug encapsulation efficiency and (**B**) loading capacity of Ce6@FDC with increasing feeding content of Ce6. (**C**) Zeta potentials of Ce6@FDC at different CTAB concentrations. (**D**) Hydrodynamic diameters of Ce6@FDC at different FDC concentrations. Data are shown as means ± S.D. (n = 3).

The Ce6@FDC nanoemulsion was fabricated through the conventional thin film hydration approach [26]. Various doses of Ce6 were added into the nanoemulsion and then the loading behavior of Ce6 in the nanoemulsion was investigated by a fluorescence spectrophotometer. As shown in Figure 2A,B, LC and EE was gradually elevated, which was proportional to the fed amounts of Ce6. The LC and EE reached 20.7% and 97.6% at 0.8 mg mL^−1^, respectively. The zeta potential of the Ce6@FDC nanoemulsion was 35.4 ± 1.2 mV at 2 mg mL^−1^ of CTAB, displaying a good colloidal stability (Figure 2C). For maximizing oxygen loading, the impact of the FDC content on the hydrodynamic size of Ce6@FDC was also investigated. It was found that the size was gradually increased while increasing the FDC content, even up to 10%. The hydrodynamic diameter of the obtained Ce6@FDC was 113.5 ± 6.7 nm at 10% *v*/*v* of FDC with good colloidal stability (Figure 2D).

**Figure 3:**
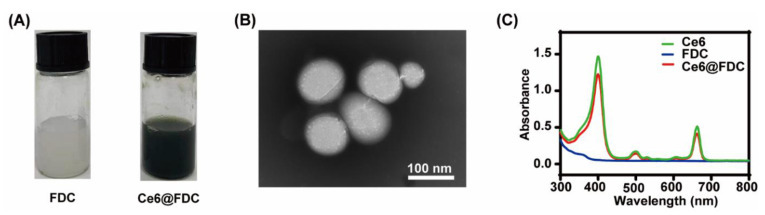
Characterization of Ce6@FDC nanoemulsion. (**A**) Photograph of FDC (left) and Ce6@FDC (right). (**B**) TEM image of Ce6@FDC. Scaler bar: 100 nm. (**C**) UV/Visible absorption spectra of Ce6, FDC, and Ce6@FDC.

Compared with the blank FDC nanoemulsion, Ce6@FDC appeared to be dark greenish following Ce6 loading (Figure 3A). A TEM image clearly indicated the uniform spherical nanoemulsion formation. The average diameter of Ce6@FDC calculated from the TEM image was 87.5 ± 0.6 nm, which was slightly smaller than that from DLS measurement (Figure 3B). This might be attributed to shrinking of Ce6@FDC in a vacuum environment. As shown in Figure 3C, Ce6@FDC exhibited intense absorption in the visible and NIR regions with characteristic peaks at 400 nm and 660 nm originating from Ce6. Collectively, we have successfully fabricated Ce6@FDC for oxygen-affordable APDT.

### 2.2. Oxygen Loading and Releasing Behavior of Ce6@FDC

**Figure 4:**
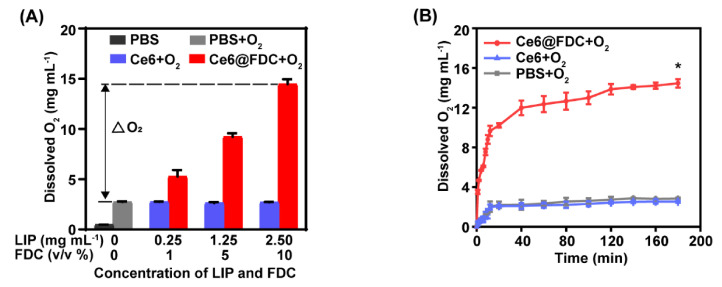
Oxygen-carrying and -releasing profile of Ce6@FDC. (**A**) The oxygen-loading capacity of Ce6@FDC. Dissolved oxygen concentration in PBS before and after adding Ce6@FDC at different concentrations. LIP: lipid. ΔO_2_: enhanced O_2_ concentration. (**B**) The oxygen-releasing behavior of Ce6 + O_2_ or Ce6@FDC + O_2_ in PBS. Data are shown as means ± S.D. (n = 3), * *p* < 0.05.

Owing to a strong oxygen-dissolving ability, FDC can be used as an excellent candidate for oxygen delivery to hypoxic biofilms. The oxygen content of Ce6@FDC + O_2_ was 5.3 ± 0.7 mg mL^−1^ and 14.5 ± 0.5 mg mL^−1^ at 1% and 10% of FDC, respectively (Figure 4A), showing a positive correlation between its oxygen-loading capacity and the FDC content. Meanwhile, we found that the lipid content has no significant impact on the oxygen-loading capacity of the Ce6 nanoemulsion, which is approximately 3 mg mL^−1^ regardless of the lipid content, which was much lower than that of Ce6@FDC. This is likely due to the marginal oxygen-loading capacity of lipids. The oxygen-release behaviors of preoxygenated Ce6 nanoemulsion (Ce6 + O_2_) and preoxygenated PBS (PBS + O_2_) were similar when they were added into the deoxygenated DI water. In contrast, the preoxygenated Ce6@FDC (Ce6@FDC + O_2_) exhibited notable oxygen release and the concentration of dissolved oxygen rapidly rose to 10.2 ± 0.5 mg mL^−1^ within 20 s in identical conditions (Figure 4B). Therefore, Ce6@FDC could serve as a preeminent nanocarrier to load and release oxygen in hypoxia regions, which can probably enhance the photodynamic effect for pathogen killing.

### 2.3. Enhanced Singlet Oxygen Generation

**Figure 5:**
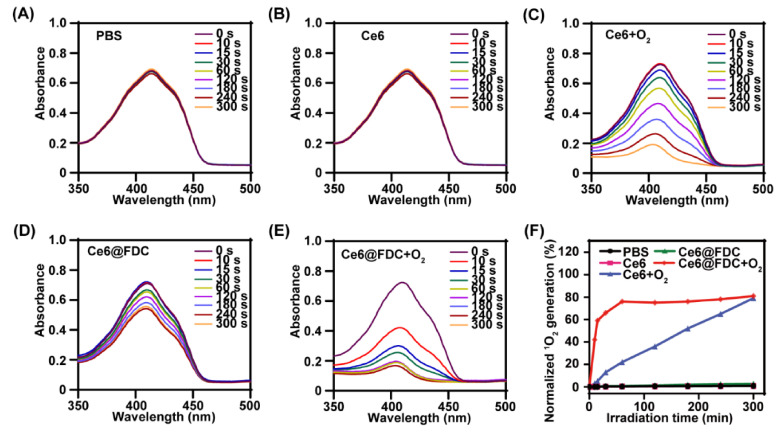
Light-triggered ^1^O_2_-generation of Ce6@FDC + O_2_ nanoemulsion. PBS (**A**), Ce6 (**B**), Ce6 + O_2_ (**C**), Ce6@FDC (**D**), and Ce6@FDC + O_2_ (**E**) were irradiated with 660 nm light at 50 mW cm^−2^ for 3 min in the presence of DPBF (100 μM) and their UV/vis spectra were monitored. (**F**) ^1^O_2_-generation percentage according to the absorbance reduction at 410 nm.

Next, the ^1^O_2_-generation capacity of Ce6@FDC + O_2_ was investigated using DPBF as a ^1^O_2_ probe upon light irradiation (50 mW cm^−2^). The bare ^1^O_2_ production of PBS and Ce6 in deoxygenated conditions again verified the oxygen-dependence of PDT (Figure 5A,B). Meanwhile, we observed notable decay of DPBF absorption at 410 nm in the Ce6 + O_2_ and Ce6@FDC + O_2_ groups, implying their effective ^1^O_2_-producing ability upon laser irradiation. However, Ce6@FDC + O_2_ exhibited significantly higher DPBF consumption rates than those of Ce6 + O_2_ and Ce6@FDC (Figure 5C–E). After irradiation for 60 s, the ^1^O_2-_generation percentage of Ce6@FDC + O_2_ achieved 80.0% vs. 21.1% in the case of Ce6 + O_2_ (Figure 5F). It suggested that Ce6@FDC + O_2_ would efficiently enhance the photosensitizing ability of Ce6 by oxygen affording, which is consistent with the results of previous oxygen-carrying PDTs [20,21,27].

### 2.4. Facilitated Bacteria Association of Ce6@FDC

**Figure 6:**
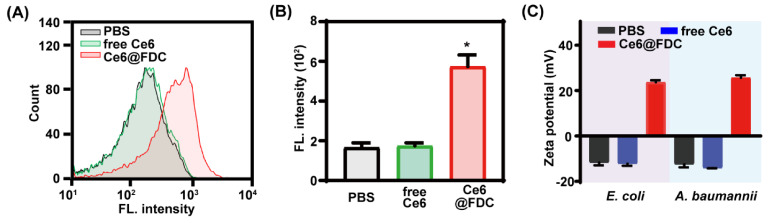
Enhanced bacterial association of Ce6@FDC nanoemulsion. (**A**) Flow cytometry analysis of *A. baumannii* treated with free Ce6 or Ce6@FDC (10 μg mL^−1^ of Ce6) for 30 min. (**B**) Corresponding fluorescence intensities of *A. baumannii* that received the indicated treatments. (**C**) Zeta potentials of *E. coli* and *A. baumannii* treated with PBS, free Ce6, or FDC@Ce6. Data are shown as means ± S.D. (n = 5), * *p* < 0.05, compared with free Ce6 group.

To investigate the interaction between bacteria and Ce6@FDC, flow cytometry analysis was performed for *A. baumannii* after treatment with PBS, free Ce6, and Ce6@FDC. As shown in Figure 6A, free Ce6 was scarcely taken up or adsorbed by *A. baumannii*, exhibiting no difference with the PBS group. In contrast, Ce6@FDC displayed notably improved association with *A. baumannii*. Quantitative analysis revealed that the fluorescence intensity of Ce6@FDC-treated *A. baumannii* was 3.3 folds higher than those treated with free Ce6 (Figure 6B). Not surprisingly, *E. coli* showed a similar association profile with Ce6@FDC as *A. baumannii* (data not shown). Zeta potential analysis revealed that free-Ce6-treated bacteria remained negative (−12.4 mV for *E. coli*; −13.2 mV for *A. baumannii*), as did the PBS-treated groups. However, the values of *E. coli* and *A. baumannii* treated with Ce6@FDC were remarkably increased to 23.7 mV and 25.63 mV, respectively, displaying a positive charge surface of bacteria (Figure 6C). These results clearly demonstrated that Ce6@FDC could strongly bind with bacteria and alter their surface properties and potently deliver Ce6 and oxygen, which is greatly advantageous for efficient PDT against antibiotic-resistant Gram-negative bacteria.

### 2.5. Photodynamic Bactericidal Performance against Antibiotic-Resistant Gram-Negative Bacteria

**Figure 7:**
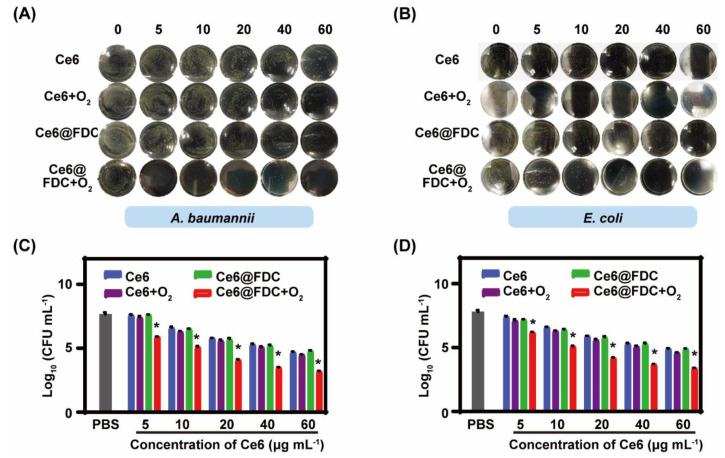
In vitro photodynamic antibacterial activity against Gram-negative strains. Photographs of plate samples of *A. baumannii* (**A**) and *E. coli* (**B**) incubated with Ce6, Ce6 + O_2_, Ce6@FDC, and Ce6@FDC + O_2_ at serial concentrations and then irradiated with a 660 nm laser (100 mW cm^−2^, 20 min). Survived numbers of *A. baumannii* (**C**) and *E. coli* (**D**) that received various treatments as indicated. Data are shown as mean ± S.D. (n = 3), * *p* < 0.05 compared to Ce6 + O_2_ and Ce6@FDC groups.

We next evaluated the photodynamic bactericidal effect of Ce6@FDC + O_2_ using the conventional spread plate method. The number of viable bacteria gradually reduced with increasing the concentration of nanoemulsion in each group, displaying a dose-dependent manner both in *A. baumannii* and *E. coli*. Almost no colonies were found on the agar plate when *A. baumannii* and *E. coli* were treated with Ce6@FDC + O_2_ (60 μg mL^−1^ of Ce6) plus light irradiation (100 mW cm^−2^ for 30 min), which was not observed in the Ce6 + O_2_ or Ce6@FDC groups under identical conditions (Figure 7A,B). For quantitative evaluation, a log of survived bacteria was calculated according to the CFU values and shown in Figure 7C,D. When *A. baumannii* was treated with Ce6@FDC + O_2_ (60 μg mL^−1^ of Ce6) plus light, the reduction in the log unit was 4.8, notably higher than 2.7 in the Ce6@FDC plus light group. Meanwhile, the analogical bactericidal effect for *E. coli* post treatments was also observed. Moreover, we observed that both the *A. baumannii* and *E. coli* treated with Ce6@FDC plus light appeared to have multiple lesions and holes, implying severe damage of the bacterial wall by oxygen-affording APDT (Figure S1, Supplementary Materials). The results demonstrated that Ce6@FDC + O_2_ exhibited potent photodynamic antibacterial efficacy in combating Gram-negative bacteria via oxygen-affording and enhanced bacteria association.

### 2.6. Potent Biofilm Ablation Ability In Vitro

**Figure 8:**
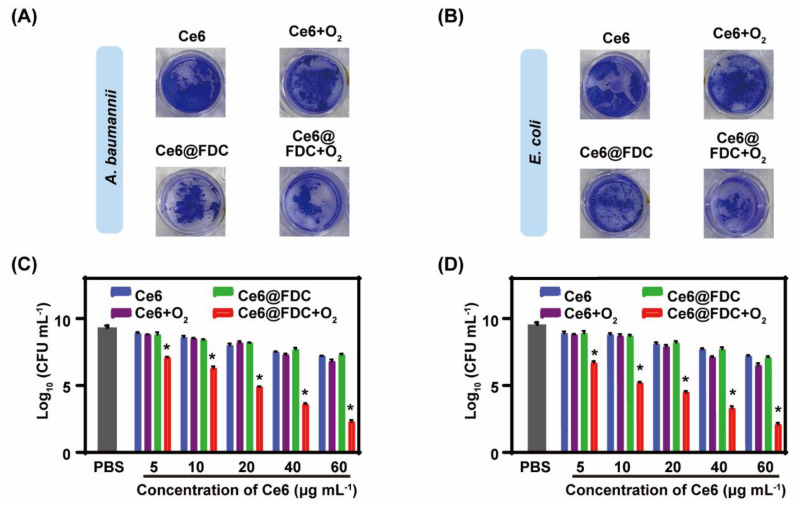
In vitro photodynamic biofilm eradiation by Ce6@FDC + O_2_ nanoemulsion. Crystal violet-stained *A. baumannii* (**A**) and *E. coli* (**B**) biofilms having received the indicated treatment for 30 min followed by irradiation (660 nm, 100 mW cm^−2^ for 20 min). Corresponding log values of viable *A. baumannii* (**C**) and *E. coli* (**D**) cells in biofilm residues by colony forming unit counting. Data are shown as means ± S.D. (n = 3), * *p* < 0.05 compared to Ce6 + O*_2_* and Ce6@FDC groups.

Encouraged by the excellent oxygen-loading capacity and photodynamic antibacterial ability of Ce6@FDC + O_2_, it is very interesting to know whether its antibiofilm performance can be enhanced. Under light irradiation, Ce6- or Ce6 + O_2_-treated biofilms appeared to be purple both in the *A. baumannii* and *E. coli* biofilms without notable differences, whereas the color of the Ce6@FDC + O_2_-treated ones was obviously lighter. Furthermore, we found that the structure of the biofilm in the Ce6@FDC + O_2_ group was severely damaged, likely due to oxygen release from the nanoemulsion (Figure 8A,B). The treated biofilms were dispersed and the survived bacteria within the biofilm were quantitated by CFU counting. The log was calculated according to the CFU values and shown in Figure 8C,D. With light irradiation, a negligible antibiofilm performance of Ce6, Ce6 + O_2_, and Ce6@FDC was found at concentrations of Ce6 from 5 to 60 μg mL^−1^. Comparatively, Ce6@FDC + O_2_ showed markedly dose-dependent antibiofilm profiles and these were much stronger than for the other groups under identical conditions. When treated with Ce6@FDC + O_2_ (60 μg mL^−1^ of Ce6) plus light, the log value of bacteria dropped to 2.3 and 2.1 for *A. baumannii* and *E. coli* biofilms, respectively, averaging five log units lower than the values for the Ce6@FDC plus light group. Taken together, Ce6@FDC + O_2_ could release oxygen after efficient association with bacteria in the biofilm, leading to the sensitization of antibiotic-resistant Gram-negative bacteria biofilm to APDT.

## 3. Materials and Methods

### 3.1. Materials

Chlorin e6 (Ce6) was purchased from Frontier Scientific Inc. (West Logan, UT, USA). 1,3-diphenylisobenzofuran (DPBF) was obtained from Sigma-Aldrich (St. Louis, MO, USA). Perfluorodecalin (FDC), lecithin, and cholesterol were purchased from Aladdin (Shanghai, China). Yeast extract, tryptone, agar, and crystal violet (CV) were purchased from Sinopharm Chemical Reagent Co., Ltd. (Shanghai, China). Distilled water (DW) used in the experiments was made from a Milli-Q Direct 16 Water Purification System (Millipore Corporation, Bedford, MA, USA) with resistivity higher than 18.2 MΩ cm^−1^. Other chemicals were supplied by Sinopharm Chemical Reagent Co., Ltd., China, and used as received. *E. coli* was purchased from Guangdong Microbial Culture Collection Center (Guangzhou, China) and *A. baumannii* was kindly supplied by Dr. Yishan Zheng at The Second Hospital of Nanjing.

### 3.2. Preparation of Ce6@FDC Nanoemulsion

Ce6@FDC nanoemulsion was fabricated by ultrasonic emulsification according to previous literature [16,21]. In brief, Ce6 was dissolved in methanol and lipid (lecithin: cholesterol = 4:1, *w*/*w*) was dissolved in chloroform at concentrations of 0.8 mg mL^−1^ and 2.5 mg mL^−1^, respectively, and then mixed at different mass ratios. The resultant mixture was stirred for 10 min at room temperature and the remaining solvent was completely removed by rotary evaporation. Following that, 5 mL of hexadecyltrimethylammonium bromide (CTAB, 0.02% *w*/*w* in DW) and FDC (500 μL) were added to the mixture in sequence during sonication. Free Ce6 was removed by gel filtration using a dextran gel column (PD midi Trap G-25, GE Healthcare, UK). Purified Ce6@FDC nanoemulsion was obtained and stored at 4 °C for further use. Ce6 emulsion and FDC emulsion were prepared without FDC or Ce6, respectively. Finally, oxygenated Ce6@FDC, designated Ce6@FDC + O_2_, was obtained by oxygen bubbling for 20 min.

### 3.3. Characterization

The morphology and size of Ce6@FDC nanoemulsion were observed using transmission electron microscopy (TEM) using a JSM-2100 electron microscope (JEOL, Japan) after staining with 1% phosphotungstic acid. The hydrodynamic diameters were analyzed by dynamic light scattering (DLS, Malvern Instruments, Malvern, UK) with a 10 mW He−Ne laser at 25 °C, and zeta potential values were determined by laser doppler microelectrophoresis at an angle of 22 °C using a Nano ZS90 Zetasizer (Malvern Instruments, UK). UV/vis absorption spectrum was acquired via microplate spectrophotometer (Multiskan GO, Thermo Fisher Scientific, Waltham, MA, USA). Fluorescence spectrophotometer (F7000, Hitachi, Japan) was used to acquire the fluorescence spectra. The content of Ce6 encapsulated in Ce6@FDC was calculated by absorbance at 660 nm according to the established calibration curve. The loading capacity (LC) and encapsulation efficiency (EE) of Ce6@FDC were calculated by the following equations: LC (%) = ((weight of loaded drug) /(total weight of emulsion)) × 100.; EE (%) = ((weight of loaded drug)/(weight of initially added drug)) × 100.

### 3.4. Oxygen Loading and Releasing of Ce6@FDC

For quantification of the oxygen-loading capacity, preoxygenated Ce6 and Ce6@FDC were added into deoxygenated phosphate buffered saline (PBS, 0.15 M, pH 7.4), and then the dissolved oxygen concentration of each sample was measured by portable dissolved oxygen meter at the time of oxygen equilibrium (~180 s, REX, JPF-605B, China). Meanwhile, to explore the oxygen-release behavior, Ce6 or Ce6@FDC saturated with oxygen was added into the deoxygenated PBS. The oxygen-release behavior was recorded by oxygen meter in real time. The oxygen releasing profile of oxygen-saturated PBS was measured as a negative control.

### 3.5. Light-Triggered ^1^O_2_-Generation Ability of Ce6@FDC + O_2_

Light-triggered ^1^O_2_-generation potency of Ce6@FDC + O_2_ nanoemulsion was quantitated using 1,3-diphenylisobenzofuran (DPBF) as probe [11]. For that, Ce6, Ce6 + O_2_, Ce6@FDC, or Ce6@FDC + O_2_ (5 μg mL^−1^ of Ce6) was mixed with deoxygenated dimethyl formamide (DMF) containing DPBF (100 μM) and irradiated with a 660 nm continuous wave diode laser beam (50 mW cm^−2^, Rayan Tech., Changchun, China) for 3 min. The generated ^1^O_2_ was detected with DPBF every 20 s by quantitating the reduction of its absorption at 410 nm via microplate spectrophotometer. DMF containing DPBF and deoxygenated PBS was used as a negative control.

### 3.6. Bacterial Association of Ce6@FDC

The interaction between Ce6@FDC nanoemulsion and bacteria was evaluated by flow cytometry. Briefly, *E. coli* or *A. baumannii* were cultured in Luria−Bertani (LB) broth medium at 37 °C with shaking overnight and harvested at the exponential growth phase. After being washed with PBS three times, bacteria were resuspended in PBS at 1 × 10^8^ CFU mL^−1^. Following that, bacteria suspension was treated with Ce6 or Ce6@FDC (10 μg mL^−1^, equivalent to Ce6) at 37 °C for 30 min and washed with cold PBS for semiquantitative analysis by NovoCyte 2060R flow cytometry and ACEA NovoExpress software (ACEA Biosciences inc., San Diego, CA, USA). Meanwhile, the zeta potential of bacteria treated as above was measured by Zetasizer.

### 3.7. In Vitro Photodynamic Antibacterial Activity

The photodynamic bactericidal activity of Ce6@FDC + O_2_ nanoemulsion against two Gram-negative strains, *E. coli* and *A. baumannii*, was evaluated by colony counting method. In short, bacteria (10^8^ CFU mL^−1^) were incubated with Ce6, Ce6 + O_2_, Ce6@FDC, or Ce6@FDC + O_2_ (concentration of Ce6 ranging from 0 to 60 μg mL^−1^) at 37 °C for 30 min in dark and then exposed to 660 nm laser at an intensity of 100 mW cm^−2^ for 20 min. Finally, 100 μL of bacteria suspension was diluted several times and spread onto LB agar plate and grown at 37 °C overnight. PBS was set as the negative control. All experiments were independently performed in triplicate.

The morphology of Ce6@FDC + O_2_-treated bacteria was observed by field emission scanning electron microscopy (FESEM). In brief, *A. baumannii* or *E. coli* suspension (10^8^ CFU mL^−1^) was incubated with Ce6@FDC + O_2_ (20 μg mL^−1^ of Ce6) and irradiated as indicated above. Then bacteria were fixed with 2.5% glutaric dialdehyde for 2 h. After washing with PBS, samples were dehydrated by graded ethanol (30%, 50%, 70%, 80%, 90%, and 100% for 10 min each). The fixed bacteria were coated with gold and imaged with FESEM (JEOL, Osaka, Japan).

### 3.8. In Vitro Ablation Capacity of Bacterial Biofilm

The performance of biofilm ablation was assessed via crystal violet (CV) staining assay and plate counting assay. For biofilm construction, 10 μL of *E. coli* or *A. baumannii* (10^8^ CFU mL^−1^) were added to culture dishes supplemented with 1 mL of fresh LB + 1% sucrose solid medium in 24-well plates and incubated at 37 °C under static conditions for 24 h to form fresh biofilms [28]. Then, the formed biofilms were gently washed with PBS and treated with Ce6, Ce6 + O_2_, Ce6@FDC, or Ce6@FDC + O_2_ (concentration of Ce6 ranging from 0 to 60 μg mL^−1^) at 37 °C for 30 min followed by irradiation (660 nm, 100 mW cm^−2^) for 20 min. Biofilms subjected to PBS treatment as above were used as negative control. The viable bacteria in the biofilms were detached into sterile PBS via vortexing. Obtained bacteria suspension was diluted and spread onto LB agar plates and then incubated at 37 °C for 12 h. The CFU on different plates were imaged and counted.

Meanwhile, CV staining assay was carried out for quantitative analysis of the ablation effect of Ce6@FDC + O_2_ nanoemulsion on *E. coli* or *A. baumannii* biofilm. Biofilms subjected to various treatments as above were washed with cold PBS and subsequently stained with CV at 37 °C for 30 min. The stained residual biofilms were photographed and resuspended with acetic acid (33%, *w*/*v*), and then the absorbance of supernatant was measured at 595 nm for quantitative analysis. All experiments were independently performed in triplicate.

### 3.9. Statistics Analysis

ANOVA was used to analyze all data with a Student−Newman−Keuls test for post hoc pairwise comparisons. All statistical analyses were performed using the GraphPad Prism software (version 8, Prism Software, Beijing, China). Differences with *p*-values < 0.05 were considered statistically significant.

## 4. Conclusions

In summary, we have successfully fabricated a photodynamic perfluorocarbon nanoemulsion, Ce6@FDC, which can simultaneously deliver photosensitizer and oxygen for the sensitization of antibiotic-resistant Gram-negative bacteria to APDT. Beyond the superior colloidal stability, Ce6@FDC also exhibits remarkable oxygen loading and releasing potency in an aqueous environment and excellent light-triggered singlet oxygen generation. Furthermore, we also demonstrated that Ce6@FDC could strongly bind with bacteria and alter their surface properties due to its positively charged surface, resulting in great bactericidal activity in combating antibiotic-resistant *A. baumannii* and *E. coli* compared with free Ce6. Ce6@FDC with oxygen loading also displayed a notable performance in the photodynamic eradication of Gram-negative bacteria biofilm. Therefore, Ce6@FDC, as a novel bactericidal agent enabling efficient delivery of Ce6 and oxygen, and enhanced APDT provide a new strategy for killing antibiotic-resistant Gram-negative bacteria. In future, the formulation of such a nanoemulsion needs to be further optimized for maximizing the safety and pathogen targetability, and its in vivo therapeutical efficacy is also yet to be investigated.

## Data Availability

Data is contained within the article and supplementary material.

## Supplementary Materials

The following are available online at https://www.mdpi.com/article/10.3390/ph15020156/s1, Figure S1. Field emission scanning electron microscopy images of bacteria after being treated with PBS or Ce6@FDC + O_2_ plus light (660 nm, 100 mW, 20 min). Red arrows indicate lesions and holes on bacterial wall. Scale bar: 1 µm.
