# Supplementary material for: Sensitization of Antibiotic-Resistant Gram-Negative Bacteria to Photodynamic Therapy via Perfluorocarbon Nanoemulsion
Source: Pharmaceuticals (Basel). 2022 Jan 27;15(2):156. doi: 10.3390/ph15020156 (PMC8878207; doi:10.3390/ph15020156)
Supplement: Supplementary file 1 [file pharmaceuticals-15-00156-s001.zip › pharmaceuticals-1550006-supplementary.pdf]

SUPPLEMENTARY MATERIALS

Sensitization of antibiotic-resistant Gram-negative bacteria to photodynamic therapy via perfluorocarbon nanoemulsion

Peiyuan Niu †, Jialing Dai †, Zeyu Wang, Yueying Wang, Duxiang Feng, Yuanyuan Li * and Wenjun Miao *

School of Pharmaceutical Sciences, Nanjing Tech University, Nanjing 211816, P. R. China; niupy@njtech.edu.cn (P.N.); daijl@njtech.edu.cn (J.D.); wzyd@njtech.edu.cn (Z. W.); wangyueying@njtech.edu.cn (Y.W.); fengdx@njtech.edu.cn (D.F.)

* Correspondence: liyy@njtech.edu.cn (Y.L.); miaowj@njtech.edu.cn (W.M.); Tel.: +86-25-58139399 (W.M.)

† These authors contributed equally to this paper.

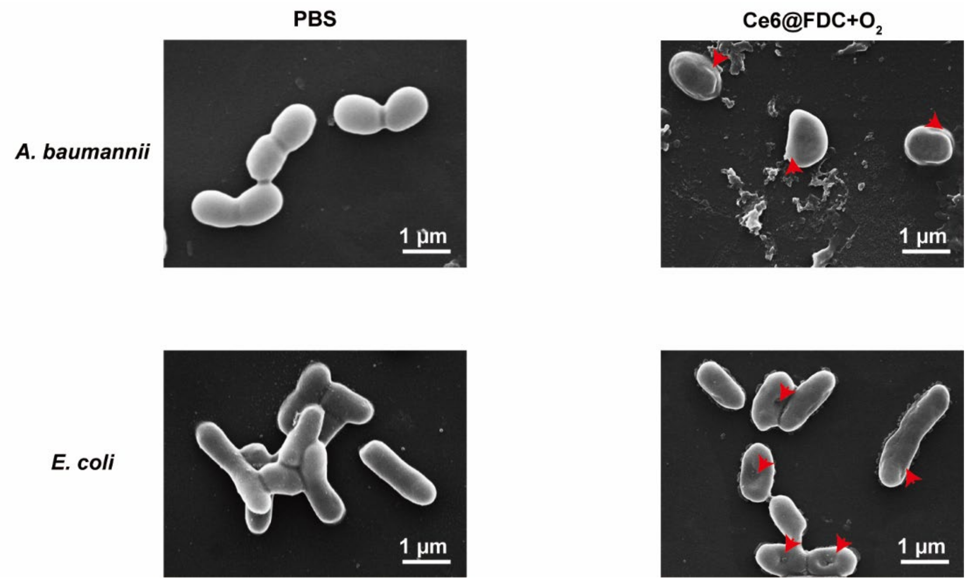

Figure S1. Field emission scanning electron microscopy images of bacteria after treated with PBS or Ce6@FDC + O_2 plus light (660 nm, 100 mW, 20 min). Red arrows indicate lesions and holes on bacterial wall. Scale bar: 1 µm.
